# Extracellular Vesicles in Environmental Toxicological Studies: Association between Urinary Concentrations of Phthalate Metabolites and Exosomal miRNA Expression Profiles

**DOI:** 10.3390/ijms25094876

**Published:** 2024-04-30

**Authors:** Paolo Cocci, Danilo Bondi, Carmen Santangelo, Tiziana Pietrangelo, Vittore Verratti, Angelo Cichelli, Giovanni Caprioli, Franks Kamgang Nzekoue, Manuella Lesly Kouamo Nguefang, Gianni Sagratini, Gilberto Mosconi, Francesco Alessandro Palermo

**Affiliations:** 1 School of Biosciences and Veterinary Medicine, University of Camerino, 62032 Camerino, Italy; paolo.cocci@unicam.it (P.C.); gilberto.mosconi@unicam.it (G.M.); 2 Dipartimento di Neuroscienze, Imaging e Scienze Cliniche, University “G. d’Annunzio” of Chieti, 66100 Chieti, Italy; danilo.bondi@unich.it (D.B.); carmen.santangelo@unich.it (C.S.); tiziana.pietrangelo@unich.it (T.P.); 3 Istituto interuniversitario di Miologia (IIM), 06132 Perugia, Italy; 4 Dipartimento di Scienze Psicologiche, Della Salute e del Territorio, University “G. d’Annunzio” of Chieti, 66100 Chieti, Italy; vittore.verratti@unich.it; 5 Dipartimento di Tecnologie Innovative in Medicina e Odontoiatria, University “G. d’Annunzio” of Chieti, 66100 Chieti, Italy; angelo.cichelli@unich.it; 6 Chemistry Interdisciplinary Project (CHIP), School of Pharmacy, University of Camerino, 62032 Camerino, Italy; giovanni.caprioli@unicam.it (G.C.); astride.kamgang@unicam.it (F.K.N.); manuella.kouamo@unicam.it (M.L.K.N.); gianni.sagratini@unicam.it (G.S.)

**Keywords:** biomarkers, endocrine disruptors, exosome-like vesicles, miRNA, pathway analysis, urine

## Abstract

Phthalates are chemical compounds, mainly used as additives in plastics, which are known to induce harmful impacts to the environment and human health due to their ability to act as hormone-mimics. Few studies have been reported on the relationship between human exposure to phthalates and the level of circulating microRNAs (miRs), especially those miRs encapsulated in extracellular vesicles/exosomes or exosome-like vesicles (ELVs). We examined the relationship of ELV-miR expression patterns and urine of adult men with five phthalate metabolites (i.e., mono isobutyl phthalate, mono-n-butyl phthalate, mono benzyl phthalate, mono-(2-ethyl-5-oxohexyl) phthalate, mono-(2-ethylhexyl) phthalate) to identify potential biomarkers and relevant pathways. We found significant positive associations which were further confirmed by multivariable analysis. Overall, our analyses showed that the Σ phthalate metabolite concentration was associated with a significant increase in the expression level of two miRs found in ELV: miR-202 and miR-543. Different pathways including cancer and immune-related responses were predicted to be involved in this relationship. Analyzing the specific downstream target genes of miR-202 and miR-543, we identified the phosphatase and tensin homolog (PTEN) as the key gene in several converging pathways. In summary, the obtained results demonstrate that exposure to environmental phthalates could be related to altered expression profiles of specific ELV-miRs in adult men, thereby demonstrating the potential of miRs carried by exosomes to act as early effect biomarkers.

## 1. Introduction

Phthalates are chemical compounds, mainly used as additives in plastics, which are known to induce harmful impacts to environment and human health [1,2,3]. These compounds have been recognized as endocrine-disrupting chemicals (EDCs) which are able to perturb hormone signaling affecting the development and reproductive system [4]. In addition, phthalates have been recently associated with metabolic diseases due to their ability to alter lipid metabolism pathways promoting adipogenesis and fat storage. There is also evidence of potential association between phthalate exposure and different types of cancer, including urothelial and prostate cancers [5,6]. People are constantly exposed to phthalates as demonstrated by levels of phthalate metabolites in the US and German populations [7,8,9]. In particular, Silva et al. [7] found detectable levels of metabolites monoethyl phthalate (MEP), monobutyl phthalate (MBP), and monobenzyl phthalate (MBzP) in >97% and mono-(2-ethylhexyl) phthalate (MEHP) in >75% of the samples. Koch et al. [9] showed that the highest urinary metabolite level was found for low-molecular-weight phthalates (e.g., mono isobutyl phthalate (MiBP), 1060 μg/g creatinine and MEP, 592 μg/g creatinine) with respect to high-molecular-weight phthalates metabolites (e.g., MEHP, 21.2 μg/g creatinine and mono-(2-ethyl-5-oxohexyl) phthalate (5oxo-MEHP), 59.6 μg/g creatinine). Ingestion, inhalation and dermal contact have proven to be important routes of exposure. In particular, personal care products, in which dimethyl phthalate (DMP), diethyl phthalate (DEP) and di-n-butyl phthalate (DBP) are used as fixatives, adhesives and solvents, are considered the most common routes of exposure to these environmental pollutants [10]. Other potential sources of phthalates are medical devices, thermal receipts, and food packaging materials [1,11]. Overall, diet, both for food and beverages, is likely to represent the predominant source of exposure to high-molecular-weight (HMW) phthalates [12,13].

Phthalates and their metabolites are usually detected in human urine that is probably the most frequently used non-invasive matrix in biomonitoring studies [14]. The occurrence of urinary phthalate metabolites is tens to hundreds of nanograms per milliliter suggesting their suitability as biomarkers of exposure [14]. Interestingly, urine has been recently demonstrated to be a source of extracellular vesicles (EVs, also currently named exosomes) which are nonometric lipid bilayer-bound particles that perform the selective transport of biomolecules such as proteins, lipids and nucleic acids, including microRNAs (miRs). These vesicles are indeed known as key mediators in cell–cell communication and are involved in promoting the transfer of genetic and biochemical information even between distant cells. EVs have been shown to reflect physiological and pathological molecular alterations in kidney, urothelial and prostate tissues suggesting their use as potential biomarkers for diseases [15].

Most recently, we demonstrated that the differential expression of metabolic and regulatory miRs, found in EVs, can be used for differentiating endurance athletes from physically inactive controls [16]. Similarly, several environmental studies have linked the alteration in miR expression profiles to the exposure to various environmental stressors. In particular, there is evidence that environmental chemicals, including plasticizers, can induce changes in miR expression, thus negatively regulating gene expression [17,18]. However, very little is known about the impact of phthalates on the circulating miR profiles, especially on the expression patterns of those miRs encapsulated in extracellular vesicles/exosomes or exosome-like vesicles (ELV). Therefore, the aim of the present study was to explore the relationship between environmental exposure to phthalates and target miRs related to ELV transport in order to achieve a better understanding of ELV-miRs as sensitive indicators of the effects of environmental exposure.

## 2. Results

### 2.1. Phthalate Metabolites

The concentration of phthalate metabolites in urine before (μg/mL) and after creatinine adjustment (μg/g creatinine) are reported in Table 1. Data were also categorized in two groups (Low and High) according to the median concentration of total phthalate metabolites. Mean and SD for urinary creatinine concentrations were 50.7 ± 7.9 mg/dL (median = 48.9 mg/dL), representative of the non-pathological function of the urinary tract. Urinary concentrations of MBP, MEHP, MiBP and MBzP were detected in 100% of subjects. On the contrary, mono-(2-ethyl-5-oxohexyl) phthalate (5-oxo MEHP) was the least prevalent (46%). Monomethyl phthalate (MMP) was not detected in any sample. The highest geometric mean levels were found for MiBP (70.5 μg/g) followed by MBP (17.9 μg/g), MEHP (6.9 μg/g), MBzP (3.4 μg/g) and lastly 5-oxo-MEHP (1.8 μg/g).

### 2.2. miR Expression from Urinary Exosomes

**Figure 1:**
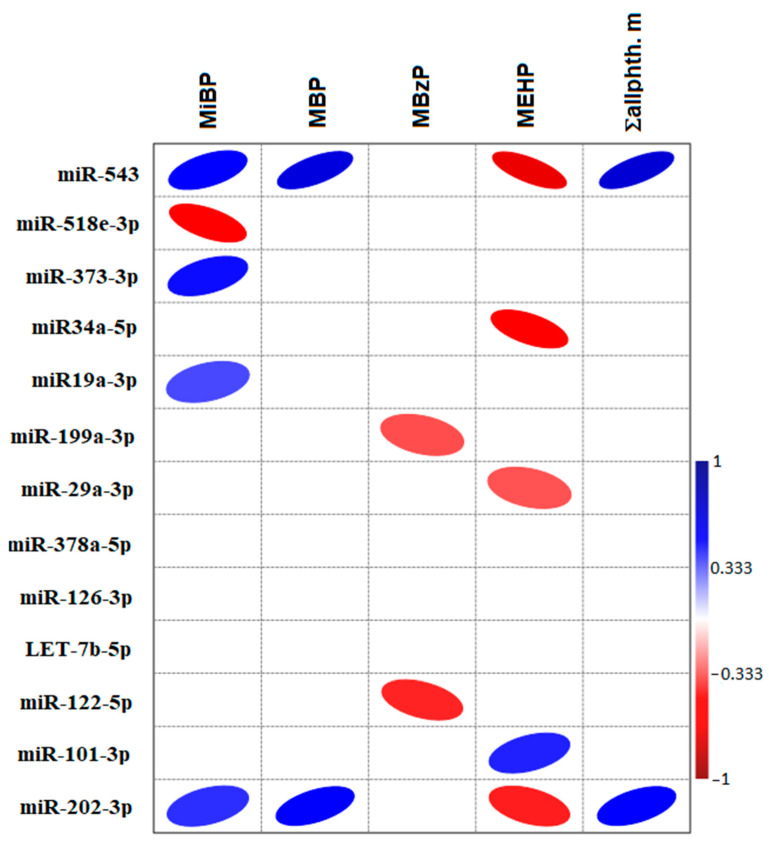
Correlation matrix between log-transformed urinary phthalate metabolites and −ΔCt of each of the 13 selected exosomal miRs. Positive correlations are displayed in blue and negative ones in red. The intensity of the color and the shape of the ellipses are proportional to the Pearson correlation coefficients. Only significant (*p* < 0.05) associations are presented in the figure.

Our analyses showed that Σ phthalate metabolite concentration was associated with a significant increase in the expression level of both miR-202 and miR-543 (Figure 1). Specifically, we found a positive significant relationship between concentrations of MiBP/MBP and both miR-543 and miR-202. Interestingly, MEHP was found to be negatively associated with miR-543, miR-202, miR-34a-5p and miR-29a-5p but positively associated with miR-101-3p. We also saw positive associations between MiBP and both miR-373-3p and miR-19a-3p. On the contrary, there was a negative relationship between MBzP and miR-199a-3p/miR-122a-5p.

After adjusting for multivariable models (Table 2), Σ phthalate metabolites together with individual metabolite levels remained significantly related with increased expression of both miR-202 and miR-543. For MiBP, the association with increased expression levels of mir-518e became less significant after including the compound concentration into Model 2. Similarly, after adjusting for creatinine, age and BMI, model 2 showed that there was no significant association between MiBP and miR-373-3p/miR-19a-3p or MEHP and miR-29a-3p expression patterns.

**Figure 2:**
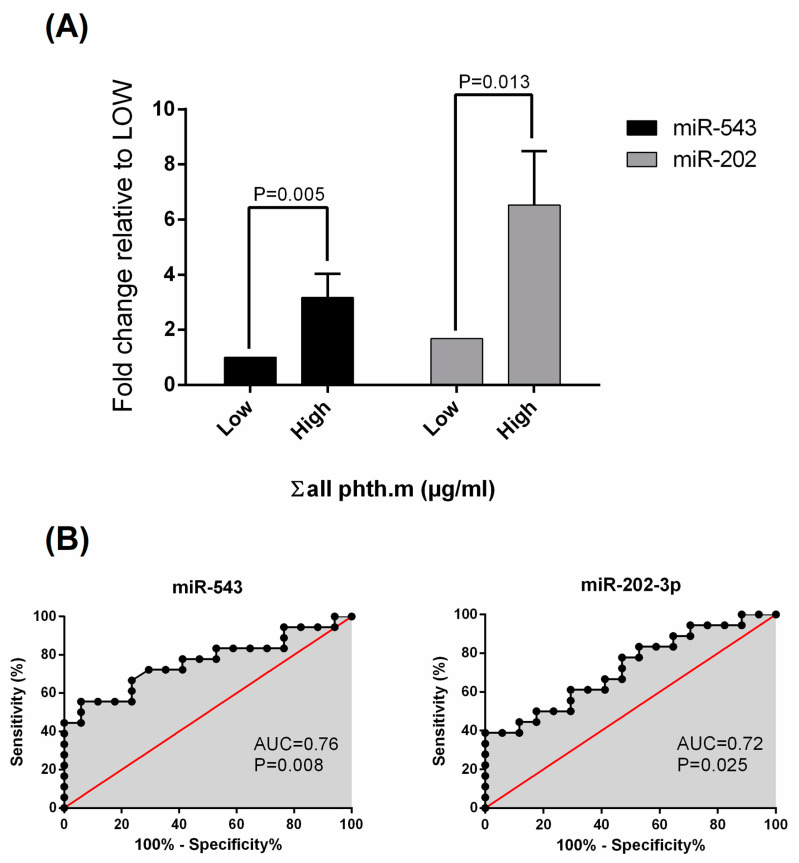
Expression levels of miR-543 and miR-202 between the two phthalate groups (low and high concentrations of total phthalate metabolites) (**A**). Biomarker potential of target miR in phthalate groups (**B**). ROC curves generated for miR-543 and miR-202 expression values as markers to distinguish low from high urinary phthalate concentrations. The area under curve (AUC), with the *p*-value, is given for each miR.

Figure 2 shows the change in miR expression levels with a 2-fold increase in median Σ phthalate metabolite urinary concentrations. The data demonstrated that an increase in concentrations of Σ phthalate metabolites in urine from the low- to high-level group would be predicted to increase miR-543 and miR-202 levels by 3.2- and 6.5-fold, respectively.

### 2.3. Computational Analysis of miR Targets

A computational analysis of miR targets cellular processes and functions associated with miR-202 and miR-543 was performed to detect the most significant pathways (Table 3).

**Figure 3:**
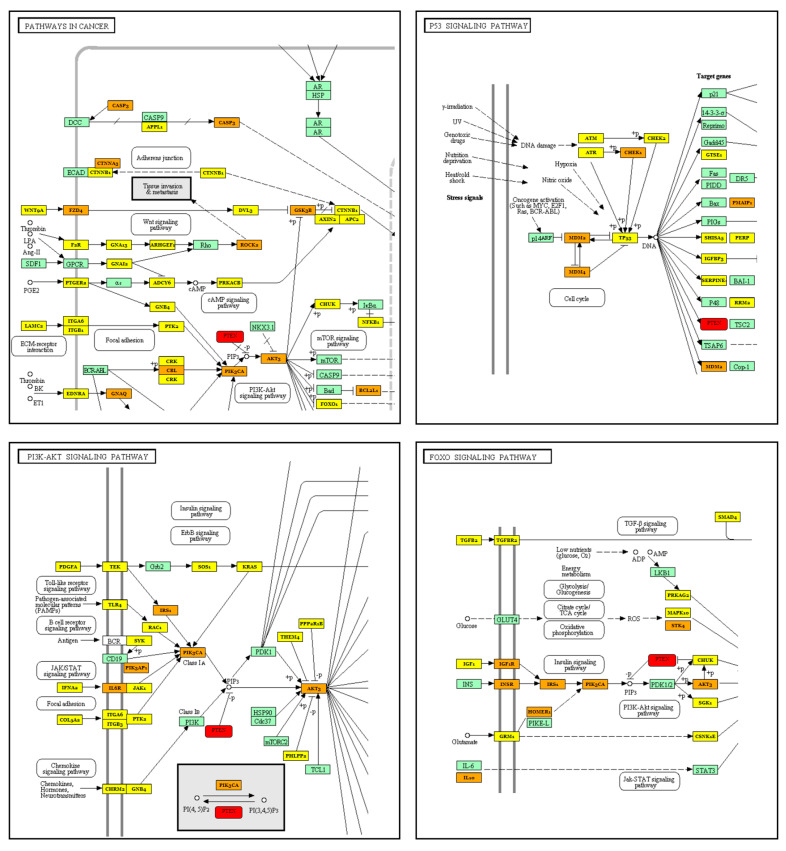
Selected KEGG pathway visualizations from DIANA-miRPath v3.0 analysis. The targeted genes are specifically labelled: green (gene not targeted by miR), yellow (gene targeted by 1 miR), orange (gene targeted by >1 miRs) and red (common gene targeted by selected miRs).

Specifically, immune-related pathways, including the phosphatidylinositol 3-kinase (PI3K)-protein kinase B (Akt), the Forkhead box, class O (FoxO), the transforming growth factor-beta (TGF-β) and the tumor necrosis factor (TNF) signaling pathways are regulated by miR-202-3p and miR-543. Analyzing the specific downstream target genes of miR-202 and miR-543, we identified the phosphatase and tensin homolog (PTEN) as the key gene in several converging pathways (Figure 3).

## 3. Discussion

All subjects had detectable concentrations of at least four urinary phthalate metabolites. Some of phthalate metabolites values were very close to the concentrations reported in US men from a previous survey [7,19]. In particular, the median value for MBP in subjects from the National Health and Nutrition Examination Survey (NHANES) was 17.7 μg/g creatinine, compared with 17.9 μg/g creatinine in our study. On the contrary, MiBP concentrations were several times higher in our study. Since the urinary concentrations of phthalate metabolites are variable, these differences may be due to the use of a spot sample rather than a 24 h sample to assess phthalates as adopted in our study. In this respect, there is scientific evidence that exposure to phthalates can also be appropriately monitored by a single urine sample [20,21]. The Pearson correlation coefficient was used to determine the relationship between log-transformed phthalate metabolite concentrations and miR expression patterns. The relationship between urinary phthalate metabolites and individual exosomal miRs was further analyzed using multivariable linear regression. Overall, our analyses showed that Σ phthalate metabolite concentration was associated with a significant increase in expression level of two miRs found in ELV: miR-202 and miR-543. Among many other associated functions, cancer and immune-related pathways are predicted to be the most targeted by these miRs. A recent study has shown the potential protective role of miR-202-3p in reducing the inflammatory response by inhibiting the Toll-Like Receptor 4 (TLR4) pathway [22]. Our findings about miR-543 targets are consistent with those described in a previous in vitro study of the involvement of miR-543-in modulating TGF-β and FoxO signaling pathways during inflammatory conditions [23]. For all these reasons, it is conceivable that an interplay between exposure to phthalates and the induction of inflammatory responses occurs through the regulation of miRs expression. In fact, it has been demonstrated that the interaction of plasticizers with several miRs might induce deep alterations in miR-mediated cell-cycle regulation of inflammation and cancer processes [24]. Chronic Bisphenol A (BPA) exposure was demonstrated to affect the miRNome in adult zebrafish causing potential adverse health outcomes including cancer. For example, miR-202 was found to be upregulated in the liver after long term BPA exposure [25]. In this latter study, a functional analysis revealed that miR-202 regulates biological processes which play pivotal roles in tumorigenesis. Furthermore, extracellular placental miR-543 may serve as biomarkers to xenobiotic exposures as its expression was significantly downregulated in pregnant women exposed to high levels of paraben metabolites and dichlorophenol metabolites. Association of metabolites with lower miR-543 may indicate potential adverse outcomes via immune system deregulation during pregnancy [26]. Interestingly, the PTEN tumor suppressor gene is a critical controller of cell proliferation and survival as well as metabolism. It has been recently demonstrated that DBP induces a reduction in mouse spermatogenesis, increases apoptosis by antagonizing the PTEN/AKT pathway and contributes to promoting DNA damage response [27]. The expression of PTEN is negatively regulated by miR-543 in colorectal cancer patients, and higher miR-543 expression promotes tumor growth [28]. In this regard, miR-202-5p also directly targets PTEN. Previous studies have indeed shown altered expression patterns of miR-202 in different cancer types [29]. In addition, it was reported that elevated levels of miR-202-5p expression affected the PTEN/PI3K/Akt pathway resulting in a deregulation of apoptotic processes and promoting cancer drug resistance. These findings indicated that miR-dependent regulation of PTEN/PI3K/Akt pathway is pivotal in mediating the effects of exposure to environmental phthalates. The obtained results suggest that environmental exposure to some phthalates is associated with altered expression profiles of specific ELV-miRs in adult men.

## 4. Materials and Methods

### 4.1. Study Population and Sampling Collection

Thirty-five males from the general Italian population were recruited for this study. The study was approved by the local ethics committee (University G. d’Annunzio Chieti-Pescara Committee Board) and was carried out in accordance with the Declaration of Helsinki. All the participants were in a healthy state without any disturbances affecting the urinary system, and they provided written informed consent before inclusion. All reported themselves as non-smokers with no smokers in their households. Urine samples were collected in the early morning in a fasting state by voluntary voiding in a sterile container, collected by study personnel, transported to the labs in a cooler with ice packs and stored at −20 °C until analysis.

### 4.2. Extraction of Urinary Exosomes

The extraction of urinary ELV by differential ultracentrifugation has been deeply described in Pietrangelo et al. [16]. Briefly, urines were centrifuged at 300× *g* (4 °C) for 10 min and then at 2000× *g* (4 °C) for 20 min. The supernatant was recovered and centrifuged (10,000× *g*) for 30 min at 4 °C. The ultracentrifugation phase included one step at 100,000× *g* (4 °C) for 70 min and a final step at 100,000× *g* for 1 h.

### 4.3. Phthalate Exposure Assessment

To avoid cross-contamination, the method of Tankiewicz et al. [30] without derivatization was applied with few modifications. As analytical standards, MMP (CAS Number 4376-18-5, MW 180.16), 5-oxo MEHP (CAS Number 40321-98-0, MW 292.33), MBP (CAS Number 131-70-4, MW 222.24), MEHP (CAS Number 111141-41-4, MW 284.315), MiBP (CAS Number 30833-53-5, MW 222.24) and MBzP (CAS Number 2528-16-7, MW 256.26) were purchased in solid form from Sigma Aldrich (Merck KGaA, Darmstadt, Germany). A 1000 ppm stock solution for each standard was prepared by dissolving the compounds in methanol (MeOH; 99.8% GC purity). Calibration standard solutions were obtained diluting standard stock solutions with MeOH. The carrier gas for the chromatographic analysis was helium (99.99% purity). To eliminate phthalates contamination, all laboratory equipment was treated twice with acetone and hexane at 100 °C for 60 min. During the analysis, we have eliminated plastic products. The quantitative analysis of monophthalates was performed using an Agilent 8890 gas chromatograph (GC) equipped with a PAL RTC 120 autosampler and an Agilent 5977B mass spectrometer (Santa Clara, CA, USA). In total, 100 μL of the urine sample was diluted in 400 μL methanol similarly to the standards, and 1 μL was injected by direct liquid injection. The injector temperature was set at 250 °C. The phthalate metabolites were isolated using an HP-5MS capillary column (30 m, 0.250 mm i.d., 0.25 μm; Agilent Technologies, Santa Clara, CA, USA). Carrier gas was used at a flow rate of 1.0 mL/min. The oven temperature was set at 60 °C for 5 min, increased to 290 °C at 20 °C/min, finally increased to 310 °C at 20 °C/min and maintained for 5 min. The ionization was obtained using an electronic ionization (EI) source. The analysis was performed in SIM mode (transfer line at 280 °C; mass analyzer at 250 °C). Phthalate metabolites were normalized by creatinine (μg/g) [19]. Creatinine has been quantified by the chromatographic method developed by Tkaczyk and Jedziniak [31].

### 4.4. Exosomal miR Extraction from Urine and Expression

Exosomal miRs were isolated from urinary exosomes using the miRNeasy Mini kit (Qiagen, Germantown, MD, USA), as described by Pietrangelo et al. [16]. MiRs were polyadenylated using a Poly(A) polymerase Tailing Kit (Diatech LabLine, Jesi, Italy). In brief, 100 ng miRs were mixed in a reaction containing Buffer (10X; Diatech LabLine, Jesi, Italy), 10 mM ATP, 2 U enzyme and were incubated at 37 °C for 1 h. The cDNA was produced from polyadenylated RNA using a universal primer (1 µM GCGAGCACAGAATTAATACGACTCACTATAGG(T)_12_VN; Eurofins Genomics, Ebersberg, Germany) and 1 µL OneScript^®^ Hot RTase, according to manufacturer’s instruction (OneScript^®^ cDNA Synthesis Kit, abm, Richmond, BC, Canada). Target miRs were chosen based on their involvement in many metabolic functions (Table 4).

For amplification reaction reagents were mixed as follows: BlasTaq 2X qPCR MasterMix (abm, Richmond, BC, Canada), Primer FW (10 μM; Table 1), Universal Primer RV (10 μM; 5′-GCGAGCACAGAATTAATACGAC-3′), diethylpyrocarbonate (DEPC)-treated water and 2 µL cDNA. Amplification reactions were set up at 95 °C for 3 min, and then 95 °C for 15 s, 60 °C for 60 s repeated for 42 cycles. The urinary exosome miR expression was calculated using miR-16-5p as a housekeeping gene [41].

### 4.5. miR Target Sequence Computational Analysis

Validated miR targets were identified using DIANA-mirPath v 3.0 (https://dianalab.e-ce.uth.gr/html/mirpathv3/index.php?r=mirpath, accessed on 13 July 2023). All miRs significantly altered following phthalate exposure were entered into the pathway analysis. Target gene intersections with all included miRs were visualized using the microT-CDS database (microT threshold: 0.5; FDR Correction: available) with statistical significance at *p* < 0.05 [42].

### 4.6. Statistical Analyses

Continuous indices were presented as the means (standard deviation) or medians (interquartile range). Preliminarily, scatter plots and Pearson correlation coefficients were used to explore the association between the expression of ELV-miRs and phthalate metabolites. Only chemicals detected in >80% of samples were included for statistical analysis. Log10 transformations were executed when the data was not normally distributed. Multivariable linear regression analysis was then performed adjusting for appropriate covariates including age, BMI and urinary creatinine concentration. Unadjusted (i.e., Model 1: crude) and adjusted (i.e., Model 2: adjusted for age, BMI and creatinine) models were used. We also categorized total phthalate metabolite concentrations into two categories: low, below the median concentration (<5.6 μg/mL), and high, above the median concentration (≥5.6 μg/mL). Finally, we used ROC methods to calculate sensitivity and specificity of miR-543 and miR-202 for predicting phthalate exposure. Statistical analyses were performed using Prism 8 GraphPad software (La Jolla, CA, USA), and *p*-values of <0.05 were considered statistically significant. Statistical significance was set at *p* < 0.05 for all analyses.

## 5. Conclusions

In this Italian male population, five phthalate metabolites were detected in their urine samples indicating exposure to four phthalate parent compounds. In our study, the urinary concentrations of phthalate metabolites were similar to that from NHANES data, except for the MiBP, which was several times higher in our samples. The main results obtained suggest that the total phthalate metabolite concentration is strongly positively correlated with two ELV- miRs: miR-202 and miR-543. These associations may reflect the risk of developing malignancies of the male urogenital tract as suggested by previous epidemiological and toxicological studies [5,6,43]. We have shown that miRs can be extracted from urinary ELVs and the ability to identify miRs associated with phthalate metabolites, thereby demonstrating the potential of ELV-miRs to act as biomarkers of environmental phthalate exposure and early biological responses. The possibility of using non-invasive samples (such as urine) to detect the effect of environmental exposure on metabolic pathways opens up intriguing perspectives both for basic science and large-sample exposome studies. However, our findings may be limited by the small number of participants and the use of a spot urine sample to assess phthalate exposure. Both are common weaknesses that can be due to the small-scale preliminary nature of this study. Regarding urine collection, there is evidence that a single spot sample may be appropriate to monitor urinary phthalate metabolites as exposure biomarkers over time (i.e., 6 months) [20,21]. In conclusion, the findings of this study may help stimulate new hypotheses on the role of ELV-miR in environmental toxicology. Additional work is underway to validate these preliminary results and provide insight into the functional association between phthalates and ELV-miR profiles.

## Figures and Tables

**Table 1:** Phthalate metabolites in urine before (μg/mL) and after creatinine adjustment (μg/g creatinine) and cut-off values within each of the two phthalate groups as described in the statistics section.

	All	Selected Categories	
Characteristics		Low (<5.6 μg/mL)	High (≥5.6 μg/mL)
Age (yr) (mean, SD)	41.97 (8.97)	45.12 (5.95)	39.00 (10.42)
BMI * (Kg/m^2^) (mean, SD)	25.56 (2.80)	25.89 (2.98)	25.24 (2.98)
Urinary phthalate concentrations (median, 25th and 75th)			
MiBP (μg/mL)	3.68 (2.97–4.27)	3.06 (2.33–3.68)	4.41 (3.83–4.89)
MBP (μg/mL)	0.86 (0.60–1.18)	0.60 (0.31–0.79)	1.19 (0.88–1.69)
MBzP (μg/mL)	0.14 (0.11–0.38)	0.16 (0.07–0.32)	0.14 (0.11–0.39)
5-oxo-MEHP (μg/mL)	0.06 (0.03–0.34)	0.02 (0.02–0.04)	0.23 (0.04–0.40)
MEHP (μg/mL)	0.58 (0.07–1.10)	0.15 (0.06–1.06)	0.82 (0.27–1.78)
Σ phthalate metabolites (μg/mL)	5.52 (4.18–7.67)	4.18 (3.92–4.47)	7.71 (6.33–8.51)
Phthalate concentrations corrected for urine creatinine (median, 25th and 75th)			
MiBP (µg/g)	74.07 (59.48–91.96)	57.87 (42.44–71.11)	91.69 (76.09–106.48)
MBP (µg/g)	18.57 (12.25–29.57)	12.03 (5.87–15.95)	29.57 (21.74–52.84)
MBzP (µg/g)	3.13 (1.96–5.16)	3.24 (1.43–4.93)	3.13 (2.57–5.22)
5-oxo-MEHP (µg/g)	1.54 (0.63–6.28)	0.50 (0.40–0.82)	3.13 (0.97–6.85)
MEHP (µg/g)	12.12 (1.73–21.57)	2.31 (1.22–17.77)	15.54 (4.43–34.79)
Σ phthalate metabolites (µg/g)	112.70 (79.98–172.0)	79.87 (72.29–86.03)	172.01 (141.55–183.51)

* BMI: body mass index.

**Table 2:** Multivariable linear regression analyses between urinary phthalate metabolites and the expression levels of exosomal miRs according to the results from the univariate analysis.

		Model 1 ^a^		Model 2 ^b^	
Compound	Outcome	B	*p*-Value	B	*p*-Value
Σ phthalate metabolites	miR-543	0.683 (0.424; 0.941)	<0.001	0.538 (0.276; 0.800)	0.001
MiBP		0.517 (0.214; 0.820)	0.001	0.373 (0.017; 0.728)	0.040
MBP		0.642 (0.370; 0.913)	<0.001	0.527 (0.282; 0.772)	<0.001
5-oxo-MEHP		−0.703 (−0.995; −0.451)	<0.001	−0.669 (−0.904; −0.434)	<0.001
MiBP	miR-518e	−0.580 (−0.868; −0.291)	<0.001	−0.485 (−0.847; −0.123)	0.010
MiBP	miR-373-3p	0.461 (0.147; 0.776)	<0.001	0.227 (−0.154; 0.607)	0.233
5-oxo-MEHP	miR-34a-5p	−0.575 (−0.864; −0.285)	<0.001	−0.449 (−0.795; −0.203)	0.002
MiBP	miR-19a-3p	0.355 (0.024; 0.686)	0.036	0.414 (−0.014; 0.842)	0.058
MBzP	miR-199a-3p	−0.346 (−0.679; −0.014)	0.042	−0.405 (−0.736; −0.075)	0.018
5-oxo-MEHP	miR-29a-3p	−0.338 (−0.671; −0.004)	0.047	−0.213 (−0.576; 0.150)	0.239
MBzP	miR-122-5p	−0.420 (−0.742; −0.100)	0.012	−0.490 (−0.831; −0.149)	0.006
5-oxo-MEHP	miR-101-3p	0.426 (0.105; −0.746)	0.011	0.458 (0.081; 0.835)	0.019
Σ phthalate metabolites	miR-202	0.513 (0.209; 0.813)	0.002	0.492 (0.148; 0.837)	0.007
MiBP		0.404 (0.080; 0.728)	0.016	0.491 (0.079; 0.904)	0.021
MBP		0.523 (0.221; 0.825)	0.001	0.491 (0.168; 0.815)	0.004
5-oxo-MEHP		−0.433 (−0.572; −0.114)	0.009	−0.439 (−0.810; −0.068)	0.022

^a^ Model 1: phthalate metabolite concentrations in urine; ^b^ Model 2: phthalate metabolite concentrations in urine + creatinine + age + BMI.

**Table 3:** Selected pathways associated with the hsa-miRs significantly altered in response to phthalate metabolites and the number of genes per pathway targeted by these miRs.

KEGG Pathway	ID	*p*-Value	Gene Count
*Colorectal cancer*	(hsa05210)	0.04	32
miR-202-3p			12
miR-543			26
*Pathways in cancer*	(hsa05200)	0.04	183
miR-202-3p			60
miR-543			157
*p53 signaling*	(hsa04115)	0.04	38
miR-202-3p			15
miR-543			34
*PI3K-Akt signaling*	(hsa04151)	0.04	159
miR-202-3p			57
miR-543			135
*FoxO signaling*	(hsa04068)	0.01	71
miR-202-3p			24
miR-543			66
*TGF-beta signaling*	(hsa04350)	0.005	46
miR-202-3p			12
miR-543			43

**Table 4:** List of forward primer sequences that are the same as the mature target miR sequences derived from the miRBase database.

miRs	Sequences	Threshold Cycle (Ct) (Mean ± SD)	References
miR-543	AAACATTCGCGGTGCACTTCTT	28.0 ± 1.4	[32]
miR-518e-3p	AAAGCGCUUCCCUUCAGAGUG	25.5 ± 1.3	[33]
miR-373-3p	GAAGTGCTTCGATTTTGGGGTGT	19.0 ± 0.7	[34]
miR-34a-5p	TGGCAGTGTCTTAGCTGGTTGT	25.1 ± 1.1	[35]
miR-19a-3p	TGTGCAAATCTATGCAAAACTGA	26.8 ± 1.3	[36]
miR-199a-3p	ACAGTAGTCTGCACATTGGTTA	27.5 ± 0.8	[37]
miR-29a-3p	TAGCACCATCTGAAATCGGTTA	23.1 ± 0.9	[35]
miR-378a-5p	CTCCTGACTCCAGGTCCTGTGT	24.6 ± 0.5	[35]
miR-126-3p	TCGTACCGTGAGTAATAATGCG	23.6 ± 0.5	[38]
LET-7b-5p	TGAGGTAGTAGGTTGTGTGGTT	27.8 ± 1.1	[35]
miR-122-5p	TGGAGTGTGACAATGGTGTTTG	20.3 ± 0.6	[35]
miR-101-3p	TACAGTACTGTGATAACTGAA	26.3 ± 1.1	[39]
miR-202-3p	AGAGGTATAGGGCATGGGAA	22.3 ± 1.7	[40]
miR-16-5p	TAGCAGCACGTAAATATTGGCG	23.4 ± 0.3	[41]

## Data Availability

The data that support the findings of this study are available from the corresponding author upon reasonable request.
